# Expression profiling of putative *Eucalyptus grandis* defence marker genes in response to treatment with methyl jasmonate and salicylic acid

**DOI:** 10.1186/1753-6561-5-S7-P96

**Published:** 2011-09-13

**Authors:** Ronishree Naidoo, Alexander Myburg, Dave Berger, Sanushka Naidoo

**Affiliations:** 1 Department of Genetics, Forestry and Agricultural Biotechnology Institute (FABI), University of Pretoria, South Africa; 2 Department of Plant Science, Forestry and Agricultural Biotechnology Institute (FABI), University of Pretoria, South Africa

## Background

*Eucalyptus* species and their hybrids encompass approximately 40% of forestry plantation area in South Africa and contribute significantly to the paper pulp industry due to their favourable wood fibre properties. Eucalypt plantation trees are affected by numerous pathogens during their lifetime, some of which can cause severe losses such as *Phytophtora* spp and *Chrysoporthe* spp. Plant defence mechanisms against pathogens is currently better understood in the model plant *Arabidopsis thaliana* where it has been shown that the salicylic acid (SA) and jasmonic acid (JA) signalling pathways enhance resistance against biotrophic and necrotrophic pathogens, respectively [1] . This process involves the up-regulation of specific defence genes which are considered to be marker (diagnostic) genes for the two signalling pathways [2,3].

## Methods

The aim of this study was to utilize the draft (8X) *E. grandis* genome sequence that has recently become available (http://eucalyptusdb.bi.up.ac.za) to identify *Eucalyptus* orthologs of defence marker genes (e.g. *PR2*; *PR3*; *PR4*; *PR5*; *LOX2*) specific for the SA and JA signalling pathways [2,3]. Bioinformatics tools were used to identify putative orthologs of these marker genes in *E. grandis* based on sequence information from other plants. This was followed by a co-phylogenetic analysis in which a neighbour-joining tree with 10 000 permutations was constructed to add confidence that the correct orthologs had been identified. In the phylogenetic tree analysis, closely related family members of a particular gene were added to increase the certainty and accuracy of selecting a specific ortholog.

The expression profile of the putative marker genes was assessed via Reverse Transcription quantitative PCR (RT-qPCR) analysis of transcript levels following treatment with various concentrations of the inducers (SA and JA) as well as different time points. This was done to confirm that the putative orthologs respond to the appropriate pathways in *Eucalyptus*. Additionally the expression profile of these putative orthologs was analyzed in response to the causal agent of *Eucalyptus* stem canker, *Chrysoporthe austroafricana*. The defence response of *Eucalyptus* to this nectrotrophic pathogen was investigated in both a tolerant (*EgrTOL*) and susceptible (*EgrSUS*) species. Changes in the level of transcript expression of the putative marker genes (*PR2; PR4; PR5; LOX3*) were assessed at three time points using RT-qPCR.

## Results and discussion

A dose response experiment of the putative marker genes was conducted with various concentrations to elucidate which would elicit the most paramount response in gene expression. It was found that amongst the tested candidates, 5mM and 100µM displayed the most significant change in gene expression for SA and JA respectively (Table 1). The specificity of the putative markers was also determined by profiling the putative marker genes with material induced by the opposing pathway, i.e SA markers were assessed with MeJA induced material.

**Table 1:** Selected results from the dose response and specificity trial

	SA (5mM)		MeJA (100μM)	
**Marker**	Exp Ratio*	**P – value**	Exp Ratio*	**P – value**

**PR2**	4.05	0.01	-0.34	0.28
**PR3**	-4.09	0.03	0.9	0.05
**PR4**	1.3	0.25	1.5	0.00005
**PR5**	0.5	0.3	1.6	0.09
**LOX2**	-4.6	0.04	0.5	0.17

*Expression ratios are represented as LOG2 values relative to the control samples

A time course experiment was done to investigate how the expression profiles of the genes respond over a period of time. This would shed light on a possible window period that one could focus on for enhancing resistance as the timing of defence is crucial in determining the outcome of a pathogen interaction. For example the *PR2* gene, a marker for the SA pathway, was shown to be drastically induced at 24hrs followed by a decline at 48hrs. This could be due to the fact that high levels of SA are toxic to the cell so the plant needs to closely monitor SA levels. On the other hand *PR4,* a marker for the JA pathway displayed a gradual increase over time beginning at 6hrs and peaking at 48hrs.

When the putative marker genes were assessed in tissue infected with *Chr. austroafricana,* it was observed that role of SA could potentially have a crucial role in determining the outcome of the infection. It is interesting to note that at two weeks there is no significant difference in lesion length between *EgrSUS* and *EgrTOL*. In *EgrTOL*, the expression level of *PR2* was significantly up-regulated at two weeks post-inoculation whereas *EgrSUS* had significantly altered levels of expression only at six weeks. However in *EgrSUS*, the level to which PR2 is induced is still lower than in *EgrTOL*. At two weeks and six weeks there is an increase in *PR4* transcript levels in *EgrSUS*. This could explain the inability of *EgrSUS* to accumulate SA due to the antagonistic relationship between the two pathways which is in accordance to what is currently known in Arabidopsis [1].

## Conclusion

The genes identified in this study were tested as a diagnostic tool for the screening of pathogen challenged eucalypt plant to determine which signaling pathway(s) were playing a role in defence against various pathogens. It was found that SA could potential play a role in enhancing resistance to *Chr. austroafricana*. Future work would involve studying the expression profile of these genes in response to various other pathogens as well as to elucidate more putative marker genes. This research provides a platform from which to expand our knowledge of plant defence in *Eucalyptus* and work towards curbing tree diseases.
